# Differential encoding of temporally evolving color patterns across nearby V1 neurons

**DOI:** 10.3389/fncel.2023.1249522

**Published:** 2023-10-18

**Authors:** Sofie Skårup Kristensen, Henrik Jörntell

**Affiliations:** Neural Basis of Sensorimotor Control, Department of Experimental Medical Science, Lund University, Lund, Sweden

**Keywords:** primary visual cortex, neurophysiology, cortical neurons, multi-electrode array, extracellular recordings, information processing, color

## Abstract

Whereas studies of the V1 cortex have focused mainly on neural line orientation preference, color inputs are also known to have a strong presence among these neurons. Individual neurons typically respond to multiple colors and nearby neurons have different combinations of preferred color inputs. However, the computations performed by V1 neurons on such color inputs have not been extensively studied. Here we aimed to address this issue by studying how different V1 neurons encode different combinations of inputs composed of four basic colors. We quantified the decoding accuracy of individual neurons from multi-electrode array recordings, comparing multiple individual neurons located within 2 mm along the vertical axis of the V1 cortex of the anesthetized rat. We found essentially all V1 neurons to be good at decoding spatiotemporal patterns of color inputs and they did so by encoding them in different ways. Quantitative analysis showed that even adjacent neurons encoded the specific input patterns differently, suggesting a local cortical circuitry organization which tends to diversify rather than unify the neuronal responses to each given input. Using different pairs of monocolor inputs, we also found that V1 neocortical neurons had a diversified and rich color opponency across the four colors, which was somewhat surprising given the fact that rodent retina express only two different types of opsins. We propose that the processing of color inputs in V1 cortex is extensively composed of multiple independent circuitry components that reflect abstract functionalities resident in the internal cortical processing rather than the raw sensory information *per se*.

## Introduction

Visual input processing *in vivo* is one of the most extensively explored aspects of neocortical function. After initial processing in retinal networks, the information is forwarded to the thalamus and the cortex where multiple studies have illustrated a variety of complex cortical representations of the visual field. Studies of the neurons in the primary visual cortex (V1) have often focused on dividing neurons into topographically organized subpopulations based on their preferences for line orientation (Ohki et al., 2006; Kondo et al., 2016) or sometimes other parameters (Palagina et al., 2017). However, many, possibly all, cells in the primary visual cortex also have preferred color inputs (Cottaris and De Valois, 1998; Conway, 2001; Johnson et al., 2001; Friedman et al., 2003; Aihara et al., 2017), but correlations between the color coding specificity and the preferred line orientation seem to be absent (Gegenfurtner, 2003). Beyond the issues of input selectivity, what kind of computations V1 cells perform on color inputs has not been extensively studied.

The computations performed by a neuron can be approximated by recording its responses across various specific inputs, if those inputs represent sensory activation patterns that can be precisely reproduced across an analysis time window where repeated presentations are made. The reproducibility is important because in the cortex, the responses of an individual neuron are not only determined by the incoming sensory input but also by multiple sources of recurrent connections (Pennartz et al., 2019; Keller et al., 2020) and the resulting activity dynamics of the network in which the neuron is embedded. In somatosensory processing, internal cortical activity dynamics have been shown to greatly impact the neuronal responses to given tactile input patterns (Norrlid et al., 2021; Etemadi et al., 2022, 2023) and can thus be expected to be an important element of neuronal encoding of sensory input in general. Such recurrent connectivity can at a global scale represent the expectation or prediction of the sensory input that is made by the brain. At a local scale, that recurrent connectivity should in theory reflect the adaptations of the cortical circuitry to the predominant spatiotemporal patterns of sensory input in combination with how dominant expectations are instantiated in the circuitry. A given sensory input may therefore induce a shift in the state in the local circuitry and thereby impact the response to subsequent given sensory inputs. Experiencing a “natural scene” may be composed of series of such local state changes. Therefore, studying the cortical responsiveness to longer duration spatiotemporal inputs can be expected to trigger multiple distributed dynamic effects in the circuitry. This can tell us something about the principles of the physiological organization of the circuitry, and the computations performed by V1 neurons. The accuracy by which V1 neocortical neurons can distinguish specific spatiotemporal patterns of color inputs (i.e., identifying the “what” of the input) would be one type of initial approximation of V1 neuron computations for color inputs.

Because the cones have different wavelength tunings and are not directly connected (Li and DeVries, 2004), inputs composed of different colors are guaranteed to result in at least partly different retina photoreceptor activations at the population level. This has an advantage, since the activation of specific photoreceptor subpopulations is otherwise difficult to control in vision neuroscience experiments, unless high resolution corrections for shifts in lens focal plane and gaze direction are implemented. Therefore, we here designed stimulations composed of temporal sequences of differently colored inputs, i.e., spatiotemporal patterns of retina photoreceptor activation. The consistent activation across different populations of photoreceptors, across repeated presentations of the specific spatiotemporal inputs, is in turn an approach to achieve high precision information on how individual neurons separate different such spatiotemporal inputs. We used multi-electrode recording arrays in the anesthetized rat to obtain simultaneous recordings from multiple neurons along the same vertical axis of the V1 cortex and applied a decoding analysis to analyze the computations performed by each individual neuron across the set of spatiotemporal visual input patterns. We find that even neurons in proximity encode these spatiotemporal inputs in different ways, suggesting that the recursive connectivity is highly diversified at the level of the local circuitry.

## Materials and methods

### Surgical procedure

Adult Sprague–Dawley rats (*N* = 4, all of male sex as the sample was too few to allow across sex comparisons, weight 306–420 g) were prepared and maintained under anesthesia in the same way as Enander et al. (2019) and Wahlbom et al. (2021). General anesthesia was induced with a mixture of ketamine/xylazine (ketamine: 40 mg/kg and xylazine: 4 mg/kg). The mixture was injected intra-peritoneally while the rat was sedated with a mixture of air and isoflurane gas (3%) for 1–2 min. To maintain anesthesia, Ringer acetate and glucose mixed with anesthetic (ketamine and xylazine in a 20:1 ratio, delivered at a rate of ~5 mg/kg/h ketamine) was continuously infused through an intravenous catheter in the right femoral vein. The catheter was inserted by making an incision in the inguinal area of the hindlimb. A small part of the skull (~2 × mm) was removed to expose the primary visual (V1) cortex. After injecting the Neuropixel probe, the exposed brain area was covered in a thin layer of agarose (1%) to prevent dehydration of the brain. To ensure a light level of anesthesia that would still prevent the animal from feeling pain, the hind paws were regularly pinched in different areas to verify the absence of withdrawal reflexes. Furthermore, the presence of desynchronized brain activity was continuously monitored. The anesthetic was chosen because it has been reported to preserve the sequential order of neuronal recruitment at short time spans in evoked responses and spontaneous activity (Luczak and Barthó, 2012). Animals were sacrificed after the end of the experiment with an overdose of pentobarbital.

### Neuropixel recordings and extraction of neurons

We recorded spiking activity across visual (V1) cortical structures with Neuropixel silicon probes. V1 recordings were made in the binocular region of V1 (V1b) where input from the two eyes converge. Coordinates for the V1b recording site were −6.5 to −7.0 mm relative to bregma and 3.5–4.2 mm lateral to the midline, contralateral to the stimulated eye.

**Figure 1:**
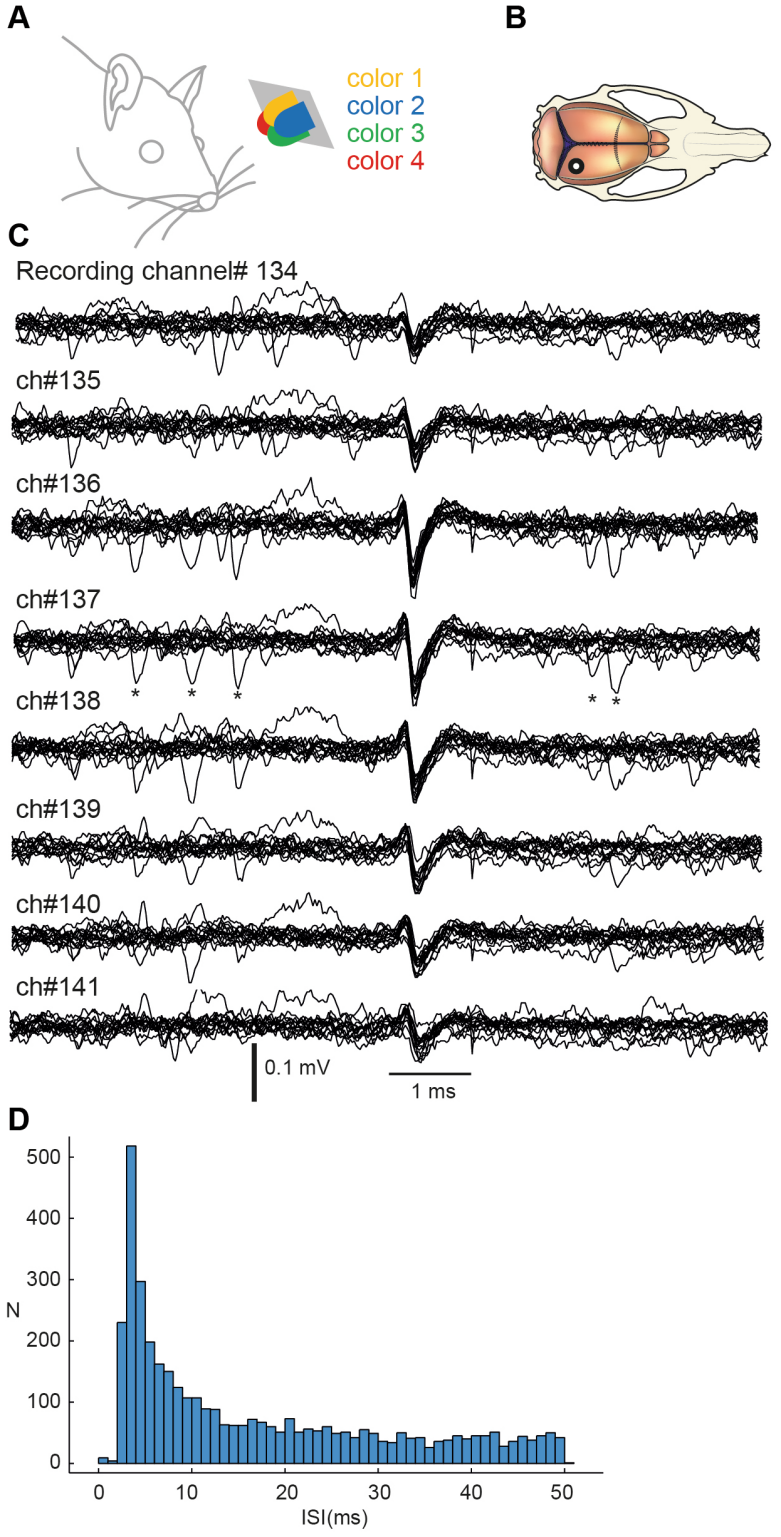
Stimulation and recording of V1 cortical neurons using Neuropixel probe multielectrode array. **(A)** Stimulation consisted of sequences of pulses from four light diodes with different colors. Diodes were smaller than shown in this figure but the distance from the left eye is approximately correctly represented. **(B)** Recordings were obtained by inserting a Neuropixel probe perpendicularly to the cortical surface with an approximate location indicated in the macroscopic view of the rat brain. **(C)** Example raw recording data from eight neighboring recording channels. Fifteen traces were aligned on the repeated occurrences of the spike of one unit. Asterisks indicate additional spike occurrences at very short intervals to the reference spike. **(D)** Interspike interval histogram for the illustrated spike.

Neuropixel recordings were processed with the Kilosort2.5 Matlab package for spike sorting. All units detected by the software were visually inspected in the open-source Python library Phy and selected based on their shape, spike frequency, and amplitude. Units with spike firing frequencies lower than 0.5 Hz were automatically deselected. Stimulation artefacts incorrectly classified as neural units were identified by their shape and then manually deselected. Units which had more than 1% of their inter-spike-intervals (ISI) within 2 ms (an example ISI histogram is shown for one sample neuron in Figure 1) were considered non-isolated units because of violation of the refractory period limiting the firing frequency of a single neuron. If the ISI plots could be improved by splitting the unit into two or more, the units were kept. If the plots did not improve, the units were deselected and not included in the analysis.

Each neuron was assigned a depth in the cortex. Neurons were first ordered by the channel number in which they displayed the largest spike signal. Cortical depth was then approximated by dividing the channel number by 100 to obtain its depth in mm.

### Stimulation

Visual stimulation was delivered as 5 ms pulses passed through a custom-made apparatus consisting of four monocolor 30mcd, 25 mA forward current, 2 V maximum supply LEDs. This type of visual stimulation has previously been shown to induce strong responses in V1 neurons (Aihara et al., 2017; Griffen et al., 2017). The LEDs were positioned at a 30° angle from the midline in the transverse plane, 4–5 cm from the left eye. The LEDs were colored red, blue, green, and yellow (shown in Figure 1A).

We used two main types of visual stimulations: multicolor pulse trains and monocolor pulse trains. Both types of stimulation were composed of four conditions each. All conditions consisted of sets of eight monocolor pulses of 5 ms duration separated by 30 ms between onsets, thus all conditions lasted for 215 ms. The monocolor stimulation conditions consisted of eight same color pulses. The multicolour stimulation conditions, i.e., the spatiotemporal patterns of visual stimulation, consisted of each of the four colors repeated twice to obtain a series of eight pulses in total, in a predefined random order. A notable exception was for one of the two blue light pulses which in all four conditions occurred as the second pulse.

**Figure 2:**
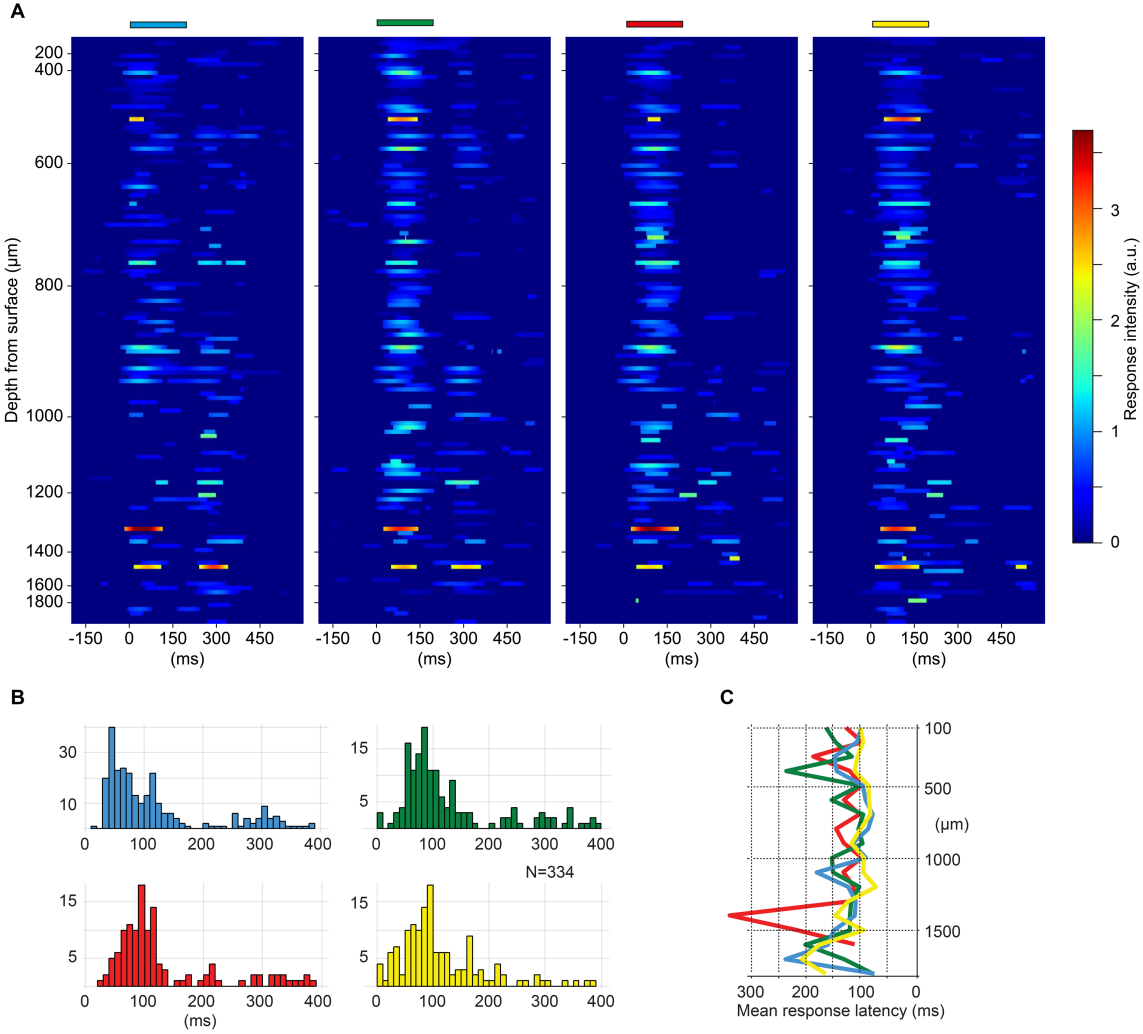
**(A)** Heatmaps of spike responses to monocolor pulse trains for all neurons (*N* = 117) in one experiment. Neurons are ordered by recording depth. The *Y*-axis indicates the recording depth, note the higher density of recorded units between 400 to 1,100 μm. The duration and color of the stimulations are indicated above each heat map. Only spike responses exceeding two SDs above mean prestimulus baseline activity are plotted. **(B)** Response latency times across the four monocolor pulse trains for all experiments (*N* = 334 neurons). Note that latency times below 20 ms are likely artefactual, due to that the function for identifying the response onset latencies includes any deviation above two SDs. **(C)** Depth plot illustrating the average latency time per color for each recording depth (100 μm bins) across all neurons.

Stimulation conditions were organized in a predefined random order in integrated stimulation protocols. A protocol consisted of 150 repetitions of each multicolor condition and 50 repetitions of each monocolor condition with a 1.5 s interval between each stimulation. Protocols were repeated three times in each experiment. Not all neurons were recorded for the entire duration of all three protocols, but all neurons were exposed to at least one protocol.

### Calculation of color responsiveness and response latency

The level of the responsiveness to individual colors and the response latencies were calculated for each neuron using peristimulus time histograms (PSTHs) of evoked responses to the monocolor pulse trains binned at 5 ms. First, a baseline activity was obtained for each individual neuron by calculating the mean spike frequency in the 200 ms prestimulus time window. If the spike frequency at any time point in the 400 ms poststimulus time window exceeded the baseline activity by two standard deviations (SDs), the neuron was considered as responding to the color. We considered a poststimulus time window of 400 ms to be potentially part of the response since we observed that some neurons would continue to respond for up to 400 ms after stimulation onset. Only very few neurons (<10) with pure inhibitory responses were observed during this inspection, and these neurons were discarded from this and all other analysis. This analysis was done to exclude neurons that did not have a detectable response to any of the colors, and also to identify neurons responding to all four colors in the decoding analysis explained below. The response latencies for individual neurons were calculated for each color separately and were defined as the time where the spike response first exceeded baseline activity by two SDs.

### Representation of the average evoked responses as a continuous function

The evoked spike responses were plotted as peristimulus time histograms (PSTHs). Kernel density estimation (KDE) was used to represent the spike responses also as time-continuous functions, overlaid in the PSTHs to minimize the impact of the discretizing step (binning) present in the PSTHs. KDEs were calculated by representing each spike event as a Gaussian distribution (kernel width 5 ms), and then averaged across the individual responses. Average KDEs were also used to visually illustrate the response intensity of different neurons to the monocolor conditions from one experiment (Figure 2A). Responses exceeding the baseline frequency (200 ms prestimulus time window of the convolved responses) by two SDs were plotted in heatmaps (Figure 2A).

**Figure 3:**
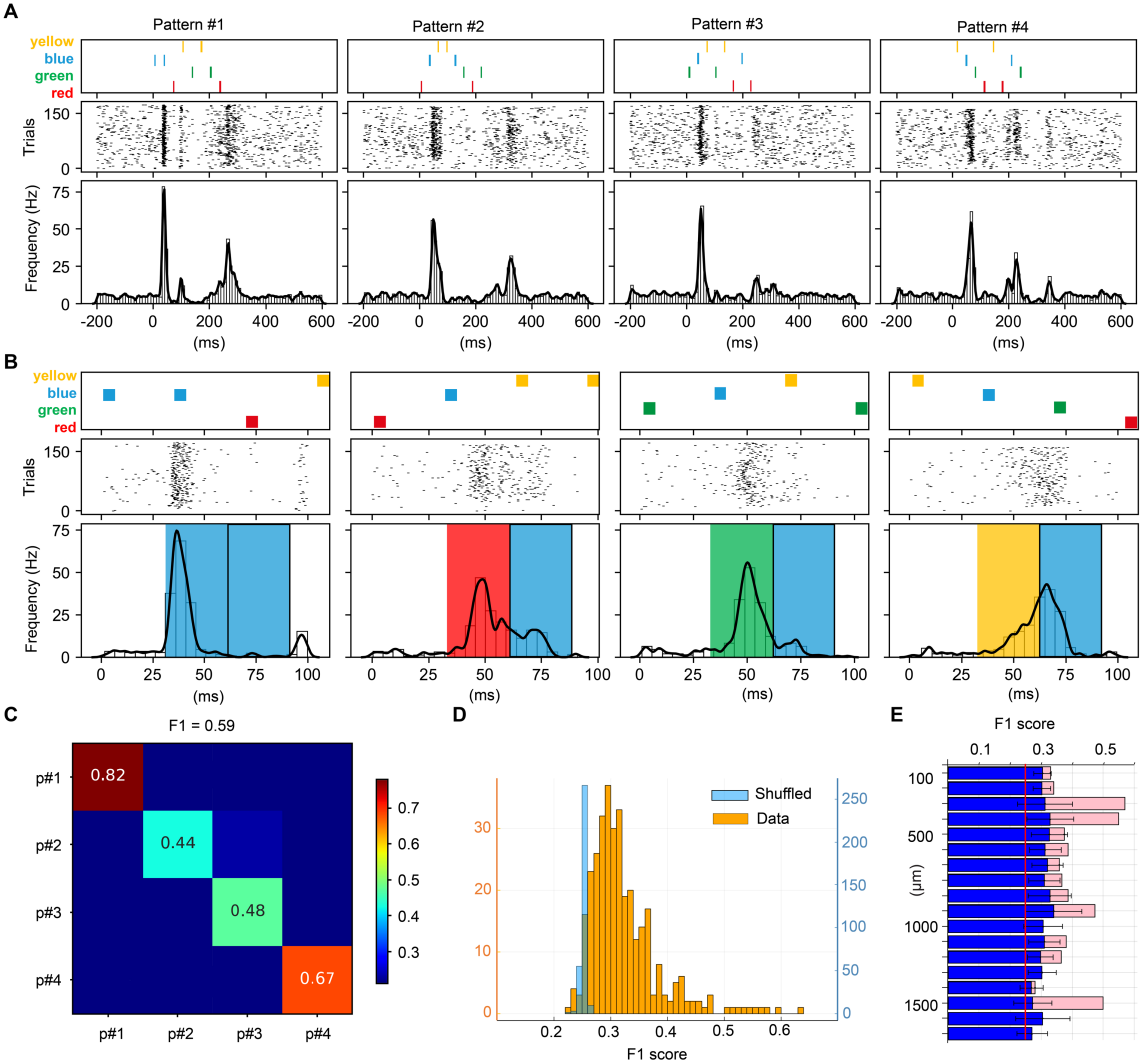
Visual multicolor pattern separation analysis for individual neurons. **(A)** Stimulation patterns and corresponding responses for a sample neuron. The responses are represented as peristimulus time histograms (PSTH bar charts) and as a kernel density estimation (KDE, solid lines) of the same responses. **(B)** Zoom-in of the first part of the stimulation patterns and the neuron responses (same neuron as in **A**). The colored boxes superimposed on the PSTHs indicate the color input for the underlying response, given the apparent shortest response latency time of 30 ms evoked by any input. Note that each pattern contained a blue pulse as the second pulse of the stimulation pattern and the figure illustrates how the color of the preceding pulse impacted the subsequent phase of the response. **(C)** Confusion matrix for the decoding of the four multicolor patterns provided for the sample neuron. The decoding performance of this unit is summarized by the *F*_1_ score measure. **(D)** *F*_1_ scores across the population of neurons, and the shuffled control data for those neurons. **(E)** Average *F*_1_ score for the pattern decoding against depth. Blue bars report the mean *F*_1_ score ± SD for the entire neuron population. Pink bars in the background report mean *F*_1_ score for units responding to all four colors.

### Spike response decoding

Our main goal was to estimate the uniqueness with which individual neurons responded to the multicolor input patterns. To this end, we analyzed the similarity between the individual spike train responses evoked by the different input conditions for each neuron, using a PCA + kNN decoding analysis which we have been using in multiple earlier papers studying cortical processing of tactile inputs (Oddo et al., 2017; Enander et al., 2019; Wahlbom et al., 2019). Superficially, this approach can be thought of as a cross-correlation analysis that quantifies the differences between average neuronal temporal response patterns evoked by different categories of input. However, rather than looking at average cross-correlations of such neuronal response patterns, which is statistically inconclusive, the PCA + kNN approach allows us to evaluate every individual response, and how well it correlates with all other responses. Then the differences between all the responses evoked by one category of input, compared to the responses evoked by other categories of input, are expressible as probabilities. Also an advantage compared to cross-correlation approaches, rather than looking at correlations pair-by-pair, the PCA + kNN approach allow us to compare each individual response with all other types of responses at the same time, i.e., for example to what extent the responses evoked by the blue color input differs from the responses evoked by red, green and yellow, simultaneously.

In order to conduct this analysis, all spike trains were first convolved into a time-continuous vector with a Gaussian kernel of 5 ms (same as for the KDE above). Convolved spike trains were *z*-scored before being randomly split into a test and a train data set with equivalent numbers of spike train responses evoked by each stimulation condition. We used principal component analysis (PCA) to explain 95% of the variance across the *z*-scored responses in the train set resulting in a set of principal components vectors (PCs) with the same length as the temporal responses. To determine the location of individual temporal spike responses in PC space, we calculated the score for each PC relative to each of the *z*-scored responses, i.e., the scalar product between each response temporal vector and each PC temporal vector. A *k*-nearest neighbor (*k*NN) classification algorithm using 9 neighbors was trained on the scores from the train set before classifying scores from the test set as belonging to one of the stimulation conditions. We repeated the decoding analysis 50 times, each time with a new training set. The *k*NN provides a quantitative measure of the level of uniqueness of the temporal response profiles for responses belonging to a specific condition in comparison to other categories of responses. Both the responses evoked with multicolor pulse trains and the responses evoked by the monocolor pulse trains were analyzed in this way, and the resulting data was represented in 4 × 4 matrices.

### Decoding accuracy

As a measure for the decoding accuracy, we used the *F*_1_ score (for a more easily accessible explanation, see for example https://www.v7labs.com/blog/f1-score-guide). First, precision and recall were calculated with true positives (TP), false positives (FP) and false negatives (FN):


$$\mathrm{Precision}=\frac{\mathrm{TP}}{\mathrm{TP}+\mathrm{FP}}$$

$$\mathrm{Recall}=\frac{\mathrm{TP}}{\mathrm{TP}+\mathrm{FN}}$$

With the precision and recall parameter for each of the 4 × 4 matrices, the *F*_1_-scores were calculated:


$$F_{1}=2\times \frac{\mathrm{Precision}\times \mathrm{Recall}}{\mathrm{Precision}+\mathrm{Recall}}$$

As a control, we repeated the decoding analyses with shuffled stimulation condition labels.

### Population decoding analysis

To explore the uniqueness of the spike responses to the multicolor pulse train inputs between neurons, we performed a decoding analysis for selected groups of neurons recorded at neighboring channels. Only neurons responding to all four colors were used in this analysis and the analysis was performed separately for separate experiments. Neurons were first ordered by the channel number in which they displayed the largest spike signal. Then we grouped neurons in sets of four neurons based on channel nearness principle, but only included neuron groups located within 10 channels (equivalent to neurons being located within a distance of 0.1 mm). For each group of neurons, we then performed a decoding analysis that included the differences in the responses between the neurons to the SAME input patterns, i.e., in this case the *k*NN classifier analyzed the separation of the responses to the four multicolor patterns from the four individual neurons. The result for each decoding analysis was a 16 × 16 classification matrix for that neuron group. The analysis was repeated for each neighboring neuron group identified.

To verify that spike responses to multicolor patterns would not only be separable among neighboring neurons but also among remotely located neurons, the decoding analysis was also performed for three control groups consisting of four neurons recorded from far apart channels (distance between each individual pair of neurons >0.25 mm). Only neurons responding to all four colors were included, and only neurons from the same experiment.

### Color opponency analysis

We also performed a color opponency analysis, i.e., calculated the differences in neuronal responses between different pairs of monocolor inputs. To this end we used PSTHs binned at 20 ms generated for each monocolor input. All PSTH activity below the mean plus two standard deviations of the baseline activity (calculated from the 200 ms prestimulus data) was then removed from the PSTHs. Next, the 400 ms poststimulus part of the PSTHs evoked by the individual monocolors were subtracted from each other, bin-by-bin, in six combinations (blue-yellow, blue-red, blue-green, red-yellow, red-green, yellow-green). If a neuron responded more strongly to blue than to yellow, it would have a positive value for the blue-yellow pair and vice versa. Once the color with the stronger response, and hence the resulting sign of the comparison (positive or negative) had been obtained for the color pair, we made another round of the bin-by-bin subtraction but this time keeping the absolute of the resulting value for each bin. This analysis design was motivated by that in terms of color opponency, i.e., the ability to use the signal of a neuron to deduce which color was activating the retinal cells, the timing of the response would be as important as the magnitude and by using the absolute values bin-by-bin we could also take the differences in response timing into account. Hypothetically, a neuron could have equal overall magnitude PSTHs to two different colors, but two totally different temporal response profiles, and this would not have been captured by a regular subtraction. Next, the sum of the absolute differences was divided by the sum of the values for the color that evoked the strongest response in the pair, to express the result as multiples of that response.

## Results

Our aim was to study how well neurons in the primary visual cortex could separate different spatiotemporal patterns of color stimulation (Figure 1A) and if there was any difference between neurons in this regard that depended on cortical depth. In four experiments using ketamine-xylazine anesthetized rats, we inserted a Neuropixel probe vertically against the cortical surface (Figure 1B). Units recorded between 0 and 1.9 mm of depth were considered to be cortical neurons. Each unit could be identified across multiple neighboring channels (Figure 1C). Across a set of channels, sometimes multiple identified units could therefore be present, but in those cases the units had a different waveshape signature across the neighboring recording channels. A total number of 411 neurons were identified by KiloSort, after curation, such as defining that they did not violate minimal theoretical interspike intervals (Figure 1D). Only 334 of the 411 neurons responded to at least one of the four colors. We therefore excluded the other 77 neurons from the analysis (these 77 neurons were evenly distributed across the range of recording depths). Fifty-four of the 334 neurons responded to all four colors and in one case below we analyze this population specifically, where indicated.

We first explored if there were any preferences between the neurons for specific colors that depended on depth. Figure 2A illustrates the responses of all cortical neurons recorded in one experiment, along the same recording shank, for trains of monocolor inputs. The intensity of the responses did not appear to have any striking systematic correlation with recording depth, and all four colors evoked responses across all depths. The response latency times for the four different monocolor inputs are illustrated in Figure 2B, which includes neurons recorded across all experiments. The response latency times were comparable between the four colors across the population of recorded cortical neurons. The depth distribution of the response latency times also did not systematically correlate with depth (Figure 2C).

Figure 3 illustrates the analysis of the key question asked in this investigation, if individual neurons could separate specific temporal sequences of multicolor input. The patterns used in this investigation are illustrated in Figure 3A. The raster plots and the peristimulus time histograms (PSTHs) of the responses of an example neuron are illustrated below the respective stimulation pattern. The PSTH clearly shows that this neuron responded differently to the four patterns. Figure 3B is a zoom-in to show that the responses of the neuron started to deviate immediately after the first pulse (which consisted of either one of the four colors) and the response to the second pulse (which was blue in all cases) was clearly dependent on the color of the preceding pulse. An interpretation of this observation could be that the state of the circuitry was differentially altered by the color of the first pulse and then that state change impacted the subsequent parts of the responses.

In order to quantify the differences between all the individual responses evoked by the four multicolor conditions, we performed a principal component analysis (PCA) on the full set of individual responses and then applied a *k*NN classifier. Figure 3C shows the decoding accuracy obtained by the *k*NN classifier across the four multicolor conditions for this example neuron. It can be seen that the individual responses were relatively specific for each pattern, where for example the responses to pattern#1 were highly accurately classified. To compare different neurons in this respect, we used the *F*_1_ score across all four patterns. Figure 3D illustrates the *F*_1_ score across all the neurons. In this case, with *F*_1_ scores calculated for four patterns, if the responses were random it would result in an *F*_1_ score of ¼, i.e., 0.25. Most of the neurons reported an *F*_1_ score higher than this theoretical chance level. However, in the case that our data would not be entirely random but instead would have some intrinsic structure, a limited set of random responses could still result in an *F*_1_ score slightly above the theoretical chance level. We therefore also used shuffling of the stimulus labels for the individual responses. The results are shown in light blue in Figure 3D. This analysis illustrated that for the vast majority of the neurons, the above chance *F*_1_ scores were true. We also analyzed whether the *F*_1_ score depended on recording depth (Figure 3E) but found no consistent correlation between *F*_1_ score and depth. This implied that there was no depth-dependence for how well individual neurons could separate the specific temporal sequences of color input, and overall, almost all neurons provided some level of decoding of the four multicolor pulse train conditions.

**Figure 4:**
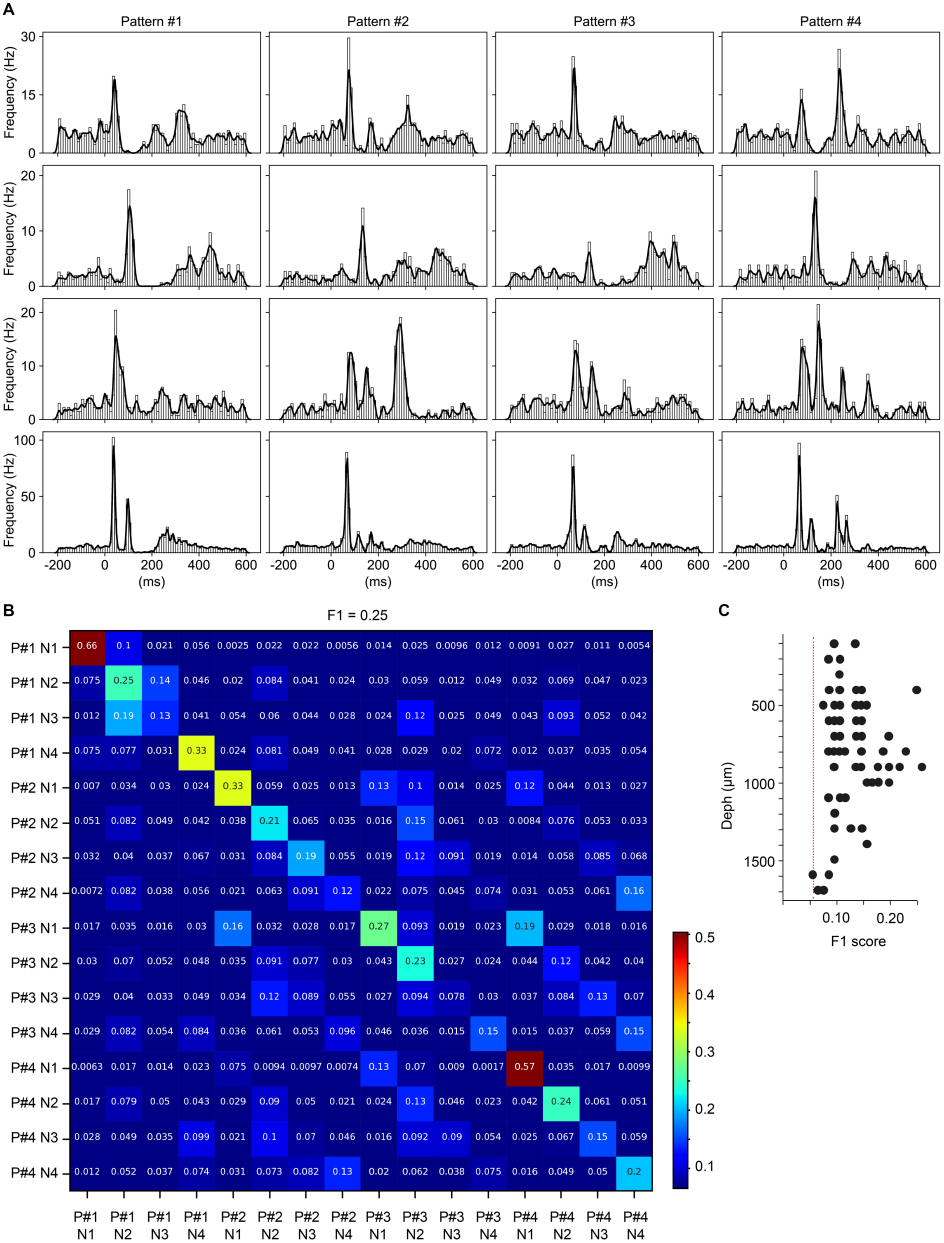
Quantification of response differences between neighboring neurons to given multicolor pulse trains. **(A)** PSTHs and KDEs of the responses of four neighboring neurons to the four multicolor conditions. **(B)** Confusion matrix of the decoding analysis of the individual responses shown in **A**, for the four neurons across the four visual patterns. **(C)** *F*_1_ scores from the decoding analyses of all groups of neighboring neurons across all experiments.

The above analysis indicated that most neurons performed well above chance with regards to the decoding of the different multicolor input conditions. In the next step, we instead explored whether neighboring neurons generated different responses to each given multicolor stimulation condition. This investigation would indicate whether neurons encoded the same input in different ways, which could give some clues to the local circuit organization by which the visual inputs were propagated to the recorded neurons. Neighboring neurons were defined as a set of four neurons recorded no more than 10 channels apart from each other. Figure 4A illustrates the responses of an example set of four neighboring neurons to the four multicolor input conditions. Comparing the average responses of the four different neurons to different input patterns (vertical axis) shows that there were relatively specific response patterns evoked in each neuron. When quantifying the level of response specificity across the set of individual responses, there was also a relatively strong diagonal in the resulting confusion matrix (Figure 4B) which confirms this notion. In the example set of four neurons, the *F*_1_ score was 0.25 (chance level = 0.0625), showing that this set of neurons had individual responses that were relatively unique, across all four stimulation conditions. Across the population of neurons, all neuron sets except one were found to provide above chance level *F*_1_ score (Figure 4C), which indicates that neighboring neurons as a rule displayed responses that to some extent were unique to each other. Figure 4C also illustrates that this inter-neuron response specificity did not correlate well with depth, although there was some tendency for neuron sets between 0.85–1.0 mm (approximately corresponding to layer IV and upper layer V) to have the highest response specificity.

We also compared sets of non-neighboring neurons, defined as neurons being located 0.25 mm away from each other, resulting in a total distance range of 1 mm for the four neurons. In these tests (*N* = 10 quadruples of neurons), similar to the one shown in Figure 4B, the *F*_1_ scores were 0.14–0.25, i.e., similar to the values obtained for neighboring neurons.

**Figure 5:**
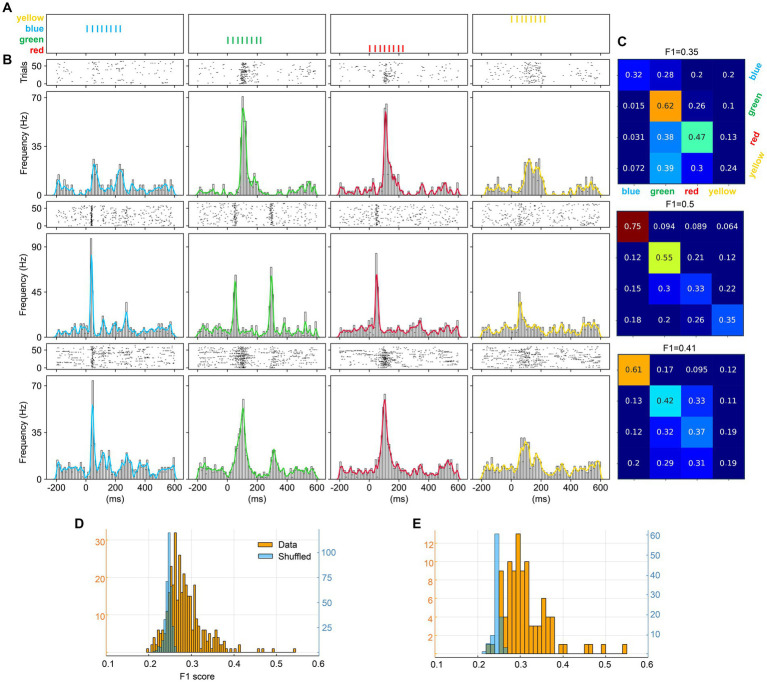
Neuronal separation analysis for the monocolor pulse trains. **(A)** The four monocolor pulse trains. **(B)** Responses from three sample neurons (N1–N3) evoked by the four monocolor pulse trains shown as PSTHs and KDEs. **(C)** Confusion matrices of the decoding analysis for the three sample neurons. **(D)** *F*_1_ scores for color separation across all individual neurons. **(E)** *F*_1_ scores for color separation including only neurons responding to all four colors.

We also examined to what extent V1 neurons could separate monocolors, using trains of monocolor pulses (Figure 5A; same stimulation conditions as used in Figure 2). The three sample neurons (Figure 5B) illustrate that each neuron responded differently to each color. The decoding analysis showed that these neurons did separate colors relatively well (Figure 5C). At the population level (Figure 5D), most neurons provided above chance *F*_1_ scores, also in comparison with the shuffled data, but compared to the multicolor input trains, the monocolor input trains provided lower decoding. For comparison, the decoding of monocolor input trains, only including neurons responding to all four colors, was somewhat stronger (Figure 5E).

**Figure 6:**
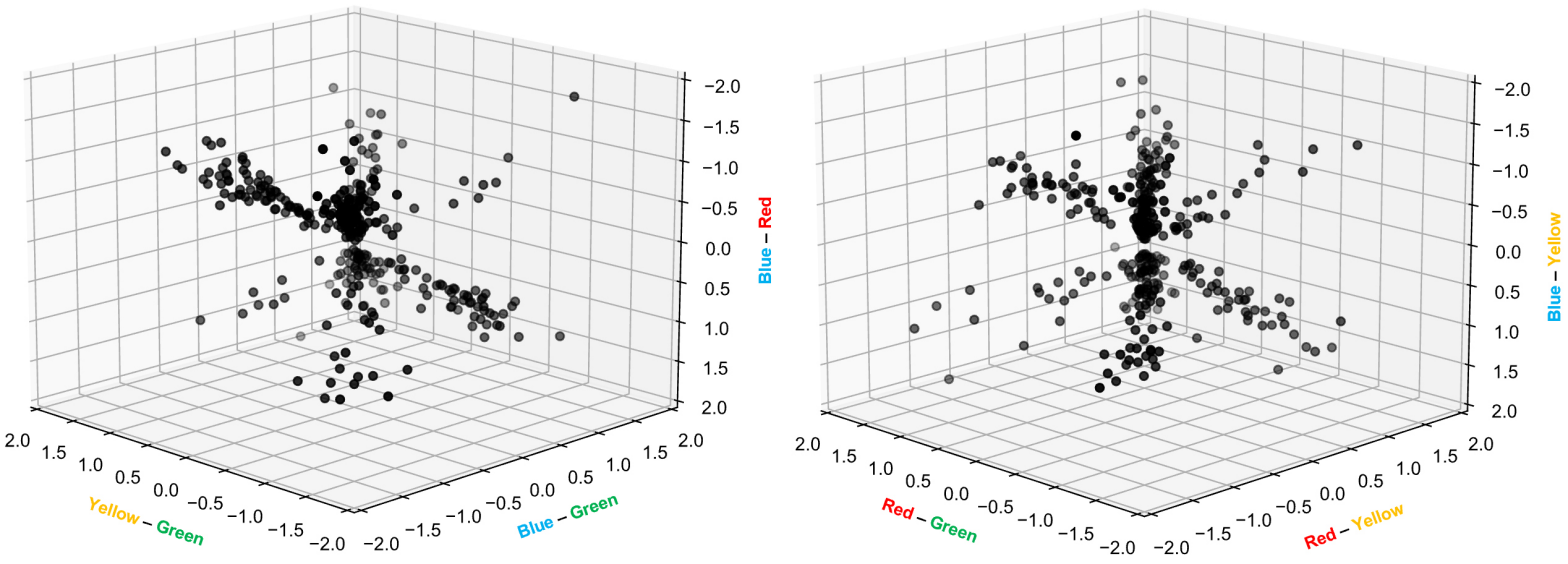
Color opponency among individual V1 cortical neurons. Relative differences in neuronal response magnitudes between the 6 pairs of colors, expressed as multiples of the reference response and presented in two separate 3D plots. The calculations were based on individual bins between 0–400 ms in PSTHs (as shown in Figure 5B) and included only bin values exceeding 2 standard deviations of the baseline activity. Note that the hue of grey indicates the depth of the data point in the 3D space.

As can also be noted from Figure 5, the variation in response magnitude across the four colors displayed different patterns across different neurons. This was interesting as differences in response magnitudes have previously been used to quantify color opponency (Yoshimatsu et al., 2021), i.e., the processing where wavelength information is extracted by comparing the neural signals from two inputs with different wavelengths. As we used four different colors as input, we could make comparisons across six different pairs of color inputs, for each neuron recorded (Figure 6). There were two remarkable observations that came out of this investigation. First, for each pairwise comparison of color input, there were a multitude of neurons that reported a major response difference, i.e., color opponency, which was somewhat surprising given that rodents express only two types of opsins in their cones (Wang et al., 2011). Secondly, no one color was standing out as evoking a stronger overall population response than any other color, as the values along each individual axis ranged from negative to positive of about equal magnitudes and as the mean value for any one color pair was close to zero [min-max ranges (mean ± standard deviation): yellow-green = −1.6 to +1.5 (0.0 ± 0.66); blue-green = −1.5 to +1.5 (−0.1 ± 0.72); blue-red = −1.7 to +1.8 (−0.1 ± 0.74); red-green = −1.5 to +1.7 (0.0 ± 0.65); red-yellow = −1.7 to +1.5 (0.1 ± 0.67); blue-yellow = −1.9 to +1.9 (0.0 ± 0.76)]. the fact that all color opponency pairs displayed both positive and negative values across the neuron population indicates that there was a true independent separation of these four color inputs, rather than some lower dimensional ordering according to some other principle such as relative differences in luminance across the four inputs.

## Discussion

We found that the spike responses of individual V1 neurons could be used to separate different spatiotemporal multicolor patterns, as well as monocolor inputs. Surprisingly, neurons along a vertical axis, across all depths, or cortical layers, were found to be similar in this respect. The neuronal separation of multicolor spatiotemporal patterns is likely indicative of that the state in the processing circuitry is gradually altered due to the specific sequence of color inputs (Figure 3). The relative neuronal specificity of the encoding of the different patterns, when the neurons were simultaneously recorded (Figure 4), showed that different neurons are likely wired into specific components of this processing circuitry.

It may seem surprising that such precise color information was represented in V1 cortex, as earlier analyses of V1 cortex in primates focused primarily on the separation of line orientation angle for the neurons in V1 cortex and instead assigns color processing to the V4 cortex. Most human patients with cerebral achromatopsia have lesions in a part of extrastriate cortex that appears equivalent to monkey area V4 (Gegenfurtner, 2003). However, cortical color processing in primates has been described as follows: “In the primary visual cortex (area V1), a large proportion of neurons respond selectively to color information. Most of these neurons also respond to variations in the brightness of visual stimuli…. In higher visual areas, neurons become more selective in their color tuning and respond only to a small range of colors” (Gegenfurtner, 2003). In fact, in the same review it is shown that neurons in V1 (and V2) of the awake macaque are rarely selective for only one color, and commonly respond to inputs of multiple colors. This is in line with the findings of Cottaris and De Valois (1998) for V1 neurons in the awake macaque. Studies in mice also indicate that V1 neurons have multicolor responses, although some degree of color selectivity is observable (Aihara et al., 2017). These findings are hence well in register with our basic observations (Figure 2A).

However, the point of the present paper was to show to what extent the V1 neurons would also be able to separate different sequences of multicolor inputs. This would be an indicator of the nature of the processing structure in the network that recursively feeds the sensory input to the population of cortical neurons. The underlying rationale is that the preceding series of inputs will alter the state of the circuitry, hence modifying the physiological structure of the recurrent activity, so that the response to a given color pulse would alter merely due to impact that the spatiotemporal structure of that series would have on the temporal evolution of the state of the cortical circuitry. This is what is illustrated in detail in Figure 3B, and overall the results of Figures 3, 4 illustrates a specificity of the encoding for different multicolor input patterns at the neuronal level as well as a specificity of encoding for each individual neuron across different multicolor input patterns.

Hence, the strongest indication of a circuitry organization that we found was the fact that neighboring neurons tended to differentially encode the same inputs (Figure 4). We have previously found a similar principle to apply for neurons engaging in processing of spatiotemporal tactile input patterns (Oddo et al., 2017; Mogensen et al., 2019; Wahlbom et al., 2021). The apparent absence of any prominent layer-wise specialization [response latency times (Figure 2); individual neuron decoding (*F*_1_ score in Figure 3); differential encoding compared to neighboring neurons (Figure 4)], was also previously found for neurons engaged in somatosensory processing (Oddo et al., 2017; Enander et al., 2019). Similar findings of considerable apparent randomness in neighboring primary visual cortical neurons have previously also been reported for the mouse (Bonin et al., 2011) although in this latter case neighboring neurons in the horizontal plane rather than in the vertical axes were studied. In fact, even for line orientation, the mouse V1 do not display a clear-cut columnar organization in that neighboring neurons can have an apparent randomness in their line orientation preference (Kondo et al., 2016).

Neurons of V4 in the awake macaque were found to have a heterogenous receptive field structure in which individual “subfields” were found to be tuned to different colors (Nigam et al., 2021). Although we did not explore different visual subfields, our result of individual neurons often responding to all four colors is of course compatible with those results. A more detailed examination of that paper also shows that the average temporal response patterns of V4 neurons were different depending on what monocolor input was provided and that individual neurons could produce different such response pattern topographies across colors (Nigam et al., 2021). This aspect is in agreement with an earlier study of the average cross-color temporal response patterns of V1 neurons in the awake macaque (Cottaris and De Valois, 1998). Whereas these two studies showed that specific colors were encoded with specific average temporal response profiles by cortical neurons, our analysis instead indicated the reliability by which each individual response of a neuron encoded a specific color (Figure 5).

Note that it is widely believed that rodents are not as dependent on color vision as humans and primates. This is to some extent based on the fact that the cones of rodents, and interestingly also new world monkeys such as the marmoset, are dichromatic, i.e., they express only two types of opsins in the retina photoreceptors, as opposed to for example old world monkeys including humans which typically are trichromatic (Conway, 2007). However, behavioral experiments have shown that both mice and rats can discriminate monochromatic lights in the blue-green range from ultraviolet light (Jacobs et al., 2001; Denman et al., 2018). Other studies have shown that rodent V1 cortex can separate colors when rod inputs had been experimentally suppressed (Aihara et al., 2017; Rhim et al., 2017). Even the assumption that red light is invisible to rodents has been challenged by Niklaus et al. (2020) who demonstrated that both the dark- and light-adapted rat retina is sensitive to far-red light. And, using knock-in genetic engineering to make mouse retina photoreceptors express a red-tuned opsin, mice could be shown to achieve a similar red-green separation as humans (Jacobs et al., 2007).

Whereas the literature to a great extent has focused on characterizing the properties of the individual opsins, color vision has multiple other mechanisms at hand. The most striking example is that individual neurons of the retina network can create different combinations of individual photoreceptor inputs to dramatically increase the color information present in the retinal output (Yoshimatsu et al., 2021). Whereas such a recombination can be achieved even by the comparatively simple retinal circuitry, much more complex recombination effects can potentially be carried out by the thalamocortical network. These two factors could well be enough to explain our surprising observation (Figure 6) of widely diversified V1 cortical color opponency across all six pairs of colors (theoretical maximum for our setup using four colors) despite that the rodent has only two types of opsins. Other potential factors could be that different cones have different percentages of M-opsins and S-opsins (Wang et al., 2011), which in theory should diversify the color tuning among the cones beyond that defined by the individual opsin, as well as the fact that rods at least in the rat also do have a degree of color tuning (Wang et al., 2011) and could thereby contribute to color opponency in an advanced processing network. It should also be noted that it is theoretically conceivable that individual cortical neurons could display a higher selectivity than what could be deduced from behavioral experiments—that is what our results suggest in comparison to existing behavioral tests for rodent color vision. The resolution provided by a population of neurons need to be at least as high as required by the behavioral test but may naturally be higher than that. Also, it is not theoretically possible to design a single behavioral experiment that would exhaustively test for all possible aspects of use of color information.

### Limitations of the study

A limitation of our study is that we only made vertical tracks with the multielectrode array, i.e., all neurons we recorded from in each experiment were aligned in the vertical axis. Electrode tracks with a horizontal angle would possibly have revealed larger differences across the neuron population. However, already in our vertically oriented recordings, it was striking how different the responses of neurons in proximity (quadruples of neurons from electrodes located within 0.1 mm from each other) were (Figure 4). Another limitation was that we deliberately aimed for a global activation of the retina, and our results can hence not say anything about the spatial resolution of the color separation that we observed. Further, as we found in a previous study for the domain of cortical tactile processing (Norrlid et al., 2021), we believe that the use of anesthetics did not in principle disrupt the function of the circuitry to the extent that the phenomena of short term circuitry operation that our results indicate would be fundamentally different from its operation principle in the awake state. Ketamine anesthesia has little such disruptive effect at short time spans (in the order of 100 s of ms) as judged by the neuronal recruitment order in spontaneous brain activity and evoked responses (Luczak et al., 2009). In agreement with these observations, a previous study of neuronal recordings in the lateral geniculate nucleus comparing the anesthetized and the awake mice concluded that; “Most qualitative receptive field parameters were found to be unchanged between the two states, such as most aspects of spatial processing, but there were significant differences in several parameters, most notably in temporal processing” (Durand et al., 2016). Also other previous studies of rodent color processing used anesthetized animals (Aihara et al., 2017; Rhim et al., 2017).

### Concluding remarks

Our findings indicate that among a local population of V1 neurons processing specific spatiotemporal patterns of color input the computational properties can differ significantly. In fact, among 100’s of cortical neurons located along the vertical axes in the same experiment, the overall impression was a diversity of neuronal encoding across the population for each given input. Such differential encoding may be important to achieve a high processing capacity in the integrated network. We interpret these results to indicate that the circuitry organization for processing color inputs in V1 cortex contains prominent elements that reflect the abstract functionalities resident within the internal cortical processing, which in turn would be reflective of its physiological network structure. Different individual V1 neurons would then be connected to different aspects of that cortical processing network in a manner that apparently do not depend on cell depth and thereby, by inference, on layer location.

## Data availability statement

The raw data supporting the conclusions of this article will be made available by the authors, without undue reservation.

## Ethics statement

The animal study was approved by Ethical approval for this research was obtained in advance from the local animal ethics committee in Lund/Malmö (Ethical permit ID: M13193-2017). The study was conducted in accordance with the local legislation and institutional requirements.

## Author contributions

SK and HJ planned and designed the study, designed the analysis, and wrote the article. SK conducted the experiments and conducted the analysis. All authors contributed to the article and approved the submitted version.
